# Surface Characterisation of Dental Resin Composites Related to Conditioning and Finishing

**DOI:** 10.3390/polym13234236

**Published:** 2021-12-03

**Authors:** Liliana Porojan, Roxana Diana Vasiliu, Mihaela Ionela Bîrdeanu, Sorin Daniel Porojan

**Affiliations:** 1 Department of Dental Prostheses Technology (Dental Technology), Center for Advanced Technologies in Dental Prosthodontics, “Victor Babeș” University of Medicine and Pharmacy, Eftimie Murgu Sq. no. 2, 300041 Timișoara, Romania; sliliana@umft.ro; 2 National Institute for Research and Development in Electrochemistry and Condensed Matter, 300569 Timisoara, Romania; mihaelabirdeanu@gmail.com; 3 Department of Oral Rehabilitation (Dental Technology), Center for Advanced Technologies in Dental Prosthodontics, “Victor Babeș” University of Medicine and Pharmacy, Eftimie Murgu Sq. no. 2, 300041 Timișoara, Romania; porojan.sorin@umft.ro

**Keywords:** atomic force microscopy (AFM), resin composite materials, surface structure

## Abstract

Due to the little information related to surface processing and conditioning of resin matrix ceramic materials previous glazing, the main purpose of this in vitro study was to investigate the effect of different surface treatments on the surface morphology of different resin composite materials. Five types of resin composite CAD-CAM materials: a resin composite ceramic Vita Enamic (E) and four types of nanoparticle-filled resins, like Lava Ultimate (L), Cerasmart (C), Shofu HC (S), Hyramic (H) were taken into consideration. Specimens received the following surface treatment protocols: conventional polishing [p], polishing and glazing [pg], conditioning with CoJet [c], conditioning with CoJet and glazing [cg], sandblasting [s], sandblasting and glazing [sg], etching [e], etching and glazing [eg]. Surface roughness was analyzed for all samples and nanosurface topographic characterization was made by Atomic Force Microscopy. The highest roughness was registered for sandblasted surfaces [s], followed by tribochemical silica airborne particle abrasion [c], and etching [e]. A very strong correlated conditioning behavior of resin nanoceramic materials, like L, C and S samples was found. The microroughness decreased thus [s] > [c] > [e]. These are moderate correlated with H, and are moderate negative correlated to E, where e is more efficient. Three-dimensional images indicated visible grain boundaries after conditioning, for all materials. After polishing and glazing, surfaces became smoother. For all tested conditioning and finishing methods, surface roughness values were within clinically acceptable limits. Finishing by polishing was proved to be a good choice for all materials taken into consideration, polishing and glazing likewise, excepting Hyramic. For Enamic and Shofu HC sandblasting or tribochemical conditioning and glazing and for Hyramic polishing and glazing are not the best options, related to nanoroughness values. Referring to the nanosurface topography, for Enamic, Cerasmart and Hyramic, glazing would be the method of choice, associated with the adequate conditioning method for each material.

## 1. Introduction

Resin composite materials combine the properties of glass ceramics and resin composite for dental restorations [1,2]. It is well known that ceramic materials can achieve aesthetic restorations and are characterized to have high fracture resistance, but low material wear and tend to accelerate the abrasion of opposing teeth. Good processability and ease of intraoral repair are also great advantages of these materials compared to ceramics. However, polymers have poor color stability and lose surface polish after wear [1,3,4,5,6,7,8]. In this context, resin-composite materials are easy to mill, to process, to polish, have low abrasiveness, can be easy repaired, and different shade and translucency options are available [9]. As with all restorative nanomaterials, they are actually nanohybrids, which contain fractions of micron-sized particles. Nanoparticles along with larger particles allow a higher theoretical packing density, creating materials with fewer defects. The principal advantages of nanocomposites over other composite materials include small filler size and reduced inter-particle separation, enhanced mechanical properties, surface hardness, improved optical properties (light transmission depends on particle size), high gloss, gloss stability and excellent polishability [10,11,12,13]. Studies found a strong correlation between the surface roughness and the mechanical properties of resin composite materials [14,15]. The same study explained that high surface roughness induces pronounced grooves. Related to surface finishing, glaze firing cannot be applied because of their resin matrix components. Therefore, these materials are conventionally polished in order to obtain smooth and shiny surfaces [16], but another option is the use of a light curing glaze. Glaze materials act like sealants, to provide smoother and glossier surfaces by decreasing surface irregularities, in order to increase the wear resistance, and to improve their stain resistance [17]. Another study in literature concluded that glazing did not protect the resin composite material surfaces against surface wear [18]. Other studies in literature [19,20] focused on the various surface treatments for these materials. The findings were, that there is no universal surface treatment and further studies have to be done in this area of interest.

Roughness measurements and topography analyses are key factors in evaluating surface-oriented scientific research related to polishing and glazing. R_a_ is defined by the arithmetic mean of the heights at all locations on the surface, and R_z_ is defined by the mean distance between the peaks and valleys, measured in 10 points. To provide a reliable analysis of surface quality and topography, high-resolution surface roughness measurement techniques with non-contact profilometers are important. Resulted 3D surface topography maps and roughness parameters are useful for surface quality evaluations [16]. Atomic force microscopy (AFM) provides 3D topographical images of surface roughness at nanometer resolution. S_a_ is defined by the arithmetic mean of the heights at all locations on the surface and S_q_ is defined by the distance between the peaks and valleys of the sampled line, measured in the y direction [3,17,18,19,20]. Dental technicians perform glaze procedures for many types of indirect aesthetic restorations. However, together with the development of CAD/CAM processed materials, there is a trend for surface finishing by polishing. Clinicians can also polish, sandblast, condition, and glaze by themselves. However, there is a lack of studies related to the effectiveness of glaze materials, especially on current CAD/CAM restorative materials. Furthermore, CAD/CAM hybrid materials incorporate urethane dimethacrylate (UDMA) instead of bisphenol Aglycidyl methacrylate (Bis-GMA). UDMA shows higher degree of conversion and displays lower water sorption than Bis-GMA. Therefore, the mechanical properties of these materials are enhanced, they provided a decrease in internal defects [13,21,22]. Because assessment of the surface characteristics in order to obtain optimal surface treatments is essential, and due to the little information related to surface processing and conditioning of resin matrix ceramic materials previous glazing, the purpose of the study was to investigate the effect of different surface treatments on the surface morphology for reliable outcomes of finished surfaces.

The novelty of this study consists in studying five different types of dental resin-composite materials and the various conditioning and finishing methods. Some of the materials are quite new in the dental field and need more research in order to provide dental clinicians useful information. The null hypotheses were that the surface morphology of the resin-composite ceramic materials is not influenced by the conditioning method, and that there are no differences in surface characteristics between different finishing protocols.

## 2. Materials and Methods

Sample size was calculated using a software G*Power number 3.1.9.4 from University Kiel (Kiel, Germany). The effect size was chosen as 0.50. The calculation revealed 8 samples for each group.

Five types of resin-composite CAD-CAM Vita Enamic (VITA Zahnfabrik, Bad Säckingen, Germany) (E) and four types of nanoparticle-filled resins (Lava Ultimate, 3M ESPE, St. Paul, MN, USA) (L), (Cerasmart, GC Corporation, Tokyo, Japan) (C), (Shofu HC, Shofu, Kyoto, Japan) (S), (Hyramic Upcera, Shenyang, China) (H) were taken into consideration (Table 1). Samples were obtained by slicing the blocks in rectangular-shaped plates (1 mm thick) (n = 32 per material), resulting in 64 surfaces, polished using silicon carbide papers (600–2000 grit) and the final thickness of each specimen was checked with a digital caliper. The samples were finally manually polished using a diamond polishing paste Renfert polish all-in-one (Renfert, Hilzingen, Germany) with a low-speed handpiece. They were cleaned for 60 to 180 s with 98% ethylic alcohol and dried. Specimen surfaces of each material were then divided into 8 groups (n = 8) in terms of the applied surface treatment method: conventional polishing, polishing and glazing, conditioning with CoJet, conditioning with CoJet and glazing, sandblasting, sandblasting and glazing, etching, etching and glazing. Specimens received the following surface treatment protocols: no treatment [only polished]; 9.5% hydrofluoric acid (Yellow Porcelain Etch; Cerkamed, StalowaWola, Poland) etching for 1 min airborne-particle sandblasted with 50 µm aluminum oxide particles (Ronvig Dental, Daugaard, Denmark) at a 10 mm distance for 15 s and tribochemical silica airborne particle abrasion with 30 µm particles CoJet (3M ESPE, Seefeld, Germany) at a 10 mm distance for 15 s. The sandblasting and tribochemical silica airborne particle abrasion treatments were performed by using an airborne-particle abrasion device DENTO-PREP™ Micro blaster (Ronvig Dental, Daugaard, Denmark) at 0.25 MPa. Tribochemical silica airborne particle abrasion is a chemical surface treatment. After the surface treatment protocols, the specimens were ultrasonically cleaned and degreased with 98% ethylic alcohol. Resin Glaze Primer (Shofu, Kyoto, Japan) was applied to the ceramic surfaces for 60 s and allowed to dry, and then two thin layers of glaze Resin Glaze Liquid (Shofu, Kyoto, Japan) were applied with a soft brush, in one direction to eliminate air bubbles and were polymerized for each 180 s in a light-polymerizing device SibariSr 620 (Sirio Dental, Meldola, Italy).

### 2.1. Surface Roughness Measurements

Specimens surface roughness was analyzed in a surface profilometer Surftest SJ-201 (Mitutoyo, Kawasaki, Japan). The arithmetic average roughness (R_a_) and maximum absolute vertical roughness (R_z_) [22] measurements were tested in 5 different directions and all data were registered. The mean value of the five measurements was calculated for each surface. The sampling length was 0.8 mm, and a force of 0.7 mN was applied.

### 2.2. Nanosurface Topographic Characterization by Atomic Force Microscopy (AFM)

Samples were examined using an atomic force microscope Nanosurf Easy Scan 2 Advanced Research (NanosurfAG, Liestal, Switzerland), in noncontact mode. Values for the average nanoroughness S_a_ (nm) and amplitude of heights S_y_ (nm) were registered. AFM investigation generated a three-dimensional image of the sample surface (2.2 µm × 2.2 µm). AFM is a cantilever-based technique that utilizes a sharp tip to interrogate surfaces at resolutions below the optical diffraction limit. It is also a powerful tool for nano-mechanical probing and measurements.

### 2.3. Statistical Analysis

Statistical analyses were performed using the Analyse-it software (Analyse-it Software, Ltd., Leeds, UK). The unpaired *t*-test was used to evaluate the comparisons between the means. A *p*-value of under 0.05 was considered statistically significant. Spearman correlation was used to assess monotonic similar or dissimilar relationships (whether linear or not) between variables. It measures the strength of association between variables and the direction of the relationship. The significance was related to: 0.00–0.19 “very weak”, 0.20–0.39 “weak”, 0.40–0.59 “moderate”, 0.60–0.79 “strong”, 0.80–1.0 “very strong”. The power of the statistical test was calculated using the software IBM SPSS (IBM, New York, NY, USA).

## 3. Results

**Figure 1:**
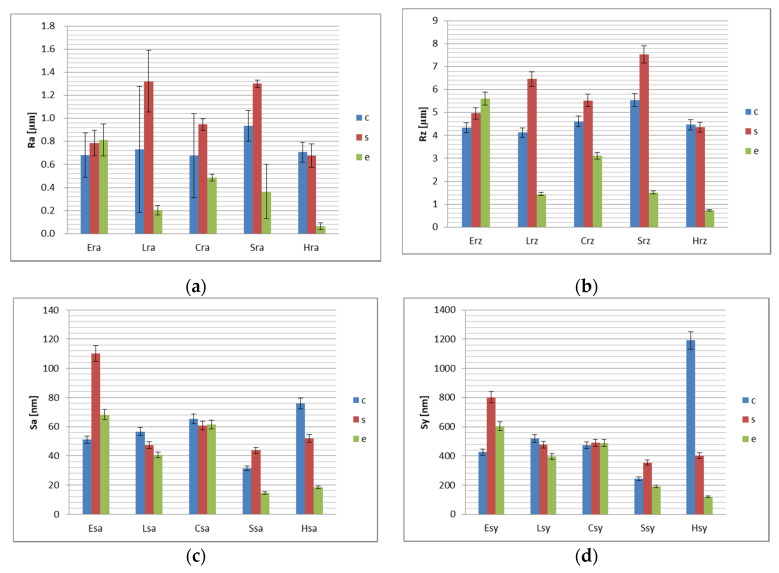
Surface roughness values of the samples, related to the conditioning method.(**a**) R_a_ (average surface roughness) for the conditioned resin-composite material samples, (**b**) R_z_ values (maximum surface roughness) for the conditioned resin-composite samples, (**c**) S_a_ (average nanoroughness) for the conditioned resin-composite samples, (**d**) S_y_ (amplitude of heights) for the conditioned resin-composite samples.

Surface roughness values R_a_, R_z_, S_a_, S_y_ for conditioned surfaces are presented in Figure 1b.

Relative to conditioning method, the results of the statistical analyses are presented in Table 2, Table 3, Table 4 and Table 5. The highest roughness was registered for sandblasted surfaces, followed by tribochemical silica airborne particle abrasion, and etching. Significant differences were found between sandblasted and etched surfaces on microroughness level and for the amplitude of heights on nanolevel. On nanolevel a very strong correlation was registered between sandblasted and etched surfaces, that means that the behavior of the materials is similar to sandblasted and etched conditioning, tribochemical silica airborne particle abrasion is less efficient for E and more efficient for H.

**Figure 2:**
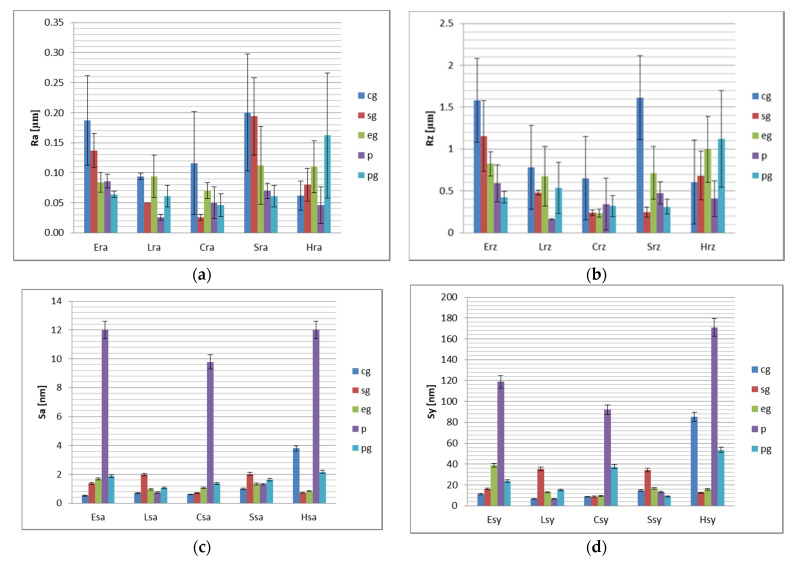
Surface roughness values of the samples, related to the finishing method. (**a**) R_a_ (average surface roughness) for the finished resin-composite samples, (**b**) R_z_ values (maximum surface roughness) for the finished resin-composite samples, (**c**) S_a_ (average nanoroughness) for the finished resin-composite samples, (**d**) S_y_ (amplitude of heights) for the finished resin-composite samples.

Surface roughness values R_a_, R_z_, S_a_, S_y_ for finished surfaces are presented in Figure 2.

As a result of materials correlations records, a very strong correlation has been measured between Lava Ultimate, Cerasmart, and Shofu, relative to microroughness (R_a_ and R_z_). A moderate correlation was registered between the listed materials and Hyramic, and a negative moderate correlation between these and Enamic. The correlation is very strong negative between Enamic and Hyramic. On nanolevel, the behavior is different, is strong correlated for L and H. Between materials, roughness values do not vary significantly, with few exceptions, for S_y_, between Lava Ultimate and Shofu, and between Cerasmart and Shofu. It can be stated that all conditioning methods are effective on all materials taken into consideration. R_a_ values are 0.745 ± 0.107 µm for tribochemical silica airborne particle abrasion, 1.006 ± 0.294 µm for sandblasted surfaces and 0.383 ± 0.287 µm for etched surfaces. S_a_ values are 56.052 ± 16.664 nm for tribochemical silica airborne particle abrasion, 62.764 ± 27.280 nm for sandblasted and 40.651 ± 24.378 for etched surfaces.

Related to the conditioning method, Lava Ultimate, Cerasmart and Shofu samples demonstrated a very strong correlated conditioning behavior, the miroroughness decreased thus sandblasted >tribochemical silica airborne particle abrasion >etched with a significant variation between sandblasted and etched. Previous materials are moderate correlated with Hyramic, to which in addition sandblasted and tribochemical silica airborne particle abrasion are close together and moderate negative correlated to Enamic, where etching is more efficient, all the conditioning methods are close together. That means the first null hypothesis: “the surface morphology of the resin-composite materials is not influenced by the conditioning method” was rejected.

Relative to finishing method, the results of the statistical analyses are presented in Table 6, Table 7, Table 8 and Table 9.

After surface finishing, significant differences were found for air abraised, glazed surfaces and polished surfaces and etched, glazed and polished samples on microroughness level and for air abraised, glazed surfaces and polished surfaces and polished, glazed and simply polished for the amplitude of heights, on nanolevel. Polished surfaces recorded significantly lower values compared to these. The cg and p are strong correlated for R_a_, that means that between materials, the behavior related to these finishing methods is similar. R_a_ values are higher for cg than for *p*, for all materials. Between materials, roughness values after finishing are not significant different, excepting R_z_ for Enamic-Cerasmart and S_a_ for Lava Ultimate-Shofu. For R_a_ it is a strong correlation for Enamic-Shofu, for them the values are polished, glazed < polished < etched and glazed < sandblasted and glazed. On nanolevel Enamic-Cerasmart are strong correlated.

After glazing R_a_ values decrease to 0.131 ± 0.053 µm for cg, 0.097 ± 0.060 µm for sandblasted and glazed and 0.094 ± 0.015 µm for etched and glazed, 0.055 ± 0.002 µm for polished, 0.078 ± 0.004 µm for polished and glazed. S_a_ values are 1.327 ± 1.394 nm for tribochemical silica airborne particle abrasion and glazed, 1.365 ± 0.647 nm for sandblasted and glazed and 1.171 ± 0.340 for etched and glazed 7.178 ± 5.689 nm for polished, 1.625 ± 0.428 nm for polished and glazed. On nanolevel the roughness of polished surfaces is higher for Enamic, Cerasmart and Hyramic.

After finishing, all measured R_a_ values are lower than 0.200 µm. For [polished] samples R_a_ values were between 0.026 and 0.086 µm, for [polished and glazed] between 0.046 and 0.064 µm, except for Hyramic (0.162 µm). For Enamic and Cerasmart glazing of polished surfaces decrease R_a_ values and significant decrease S_a_ values, for S only R_a_ values. For [etched and glazed] samples R_a_ values were between 0.070 and 0.112 µm. Related to tribochemical silica airborne particle abrasion and glazed and sandblasted and glazed the behavior is more favorable for Lava Ultimate, Cerasmart and Hyramic.

**Figure 3:**
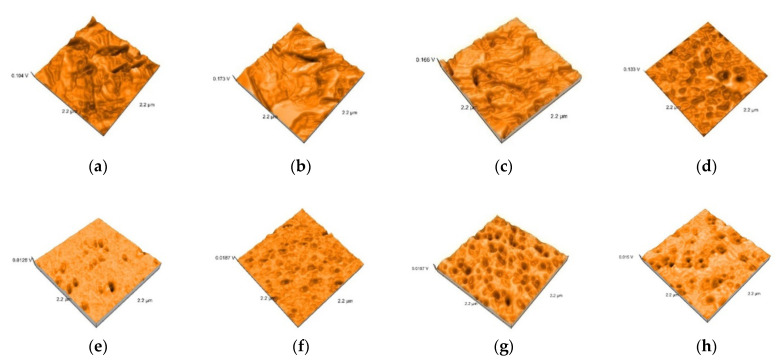
3D images of Vita Enamic specimens: (**a**) Enamic-tribochemical silica airborne particle abrasion, (**b**) Enamic-sandblasted, (**c**) Enamic-etched, (**d**) Enamic-polished, (**e**) Enamic tribochemical silica airborne particle abrasionand glazed +, (**f**) Enamic–sandblasted and glazed (**g**) Enamic etched and glazed, (**h**) Enamic polished and glazed.

**Figure 4:**
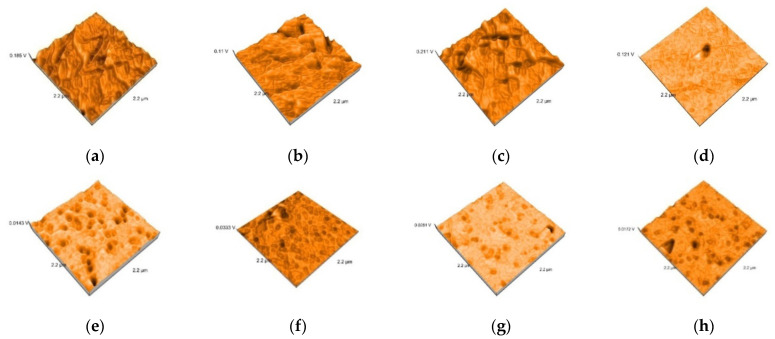
3D images of Lava specimens: (**a**) Lava Ultimate–tribochemical silica airborne particle abrasion, (**b**) Lava Ultimate sandblasted, (**c**) Lava Ultimate etched, (**d**) Lava Ultimate polished, (**e**) Lava Ultimate airborne particle abrasion and glazed, (**f**) Lava Ultimate sandblasted and glazed (**g**) Lava Ultimate etched glazed, (**h**) Lava Ultimate polished glazed.

**Figure 5:**
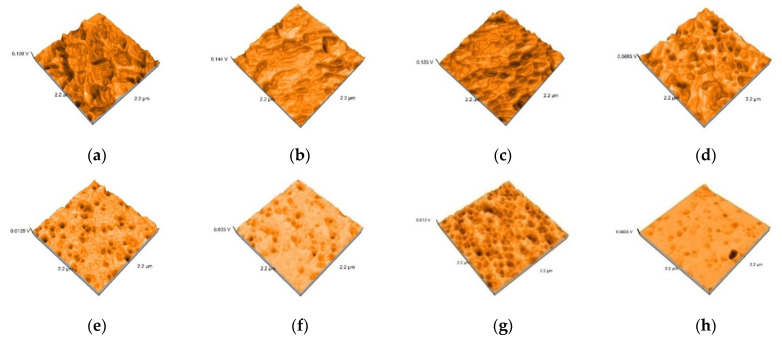
3D images of Cerasmart specimens: (**a**) Cerasmart-tribochemical silica airborne particle abrasion (**b**) Cerasmart sandblasted, (**c**) Cerasmart etched, (**d**) Cerasmart polished, (**e**) Cerasmart-tribochemical silica airborne particle abrasion and glazed, (**f**) Cerasmart sandblasted glazed, (**g**) Cerasmart etched glazed, (**h**) Cerasmart polished glazed.

**Figure 6:**
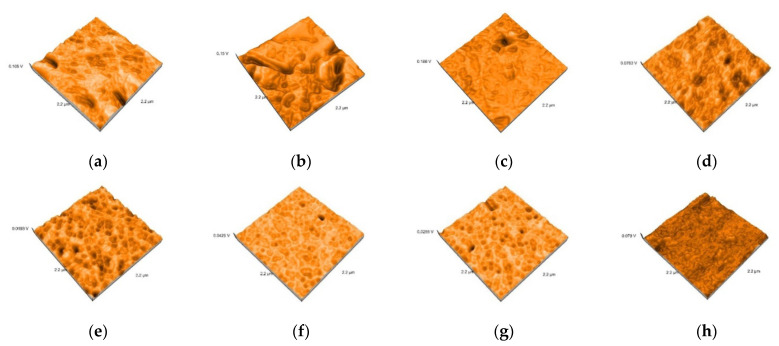
3D images of Shofu HC specimens: (**a**) Shofu tribochemical silica airborne particle abrasion, (**b**) Shofu sandblasted, (**c**) Shofu etched, (**d**) Shofu polished (**e**) Shofu tribochemical silica airborne particle abrasion and glazed, (**f**) Shofu sandblasted and glazed, (**g**) Shofu etched and glazed (**h**) Shofu polished and glazed.

**Figure 7:**
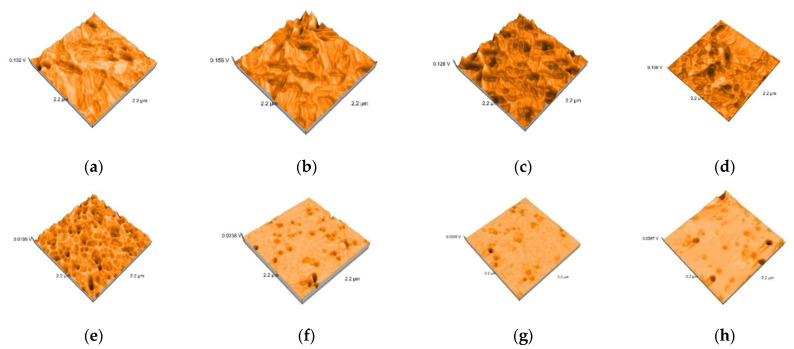
3D images of Hyramic specimens: (**a**) Hyramic tribochemical silica airborne particle abrasion, (**b**) Hyramic sandblasted, (**c**) Hyramic etched, (**d**) Hyramic polished, (**e**) Hyramic tribochemical silica airborne particle abrasion and glazed, (**f**) Hyramic sandblasted and glazed, (**g**) Hyramic etched and glazed, (**h**) Hyramic polished and glazed.

Representative AFM images of all the materials are presented in Figure 3, Figure 4, Figure 5, Figure 6 and Figure 7. The AFM images have a size of 2.2 µm^2^. The grain boundaries were clearly visible for all materials. The grains exhibited a smooth surface with a dense network of interlocking acicular branches, where interstices are filled with a compact amorphous phase. After polishing and glazing, surfaces became smoother.

## 4. Discussion

The characteristics of the material surfaces and the material compositions play an important role in obtaining favorable finishing of the surface by polishing or a thin and durable sealant layer after glazing. Therefore an improvement in bond strength is expected for microretentive surfaces and well-distributed silica content [23,24]. Because resin-composite materials combine the composition of glass ceramics and composite resins, and within this class of materials are also differences, processing procedures have to be customized. Given that there are not many such materials available on the market, surface processing investigations are welcome to be reported for each type of material.

Previous studies have suggested that pretreatments like sandblasting and tribochemical silica airborne particle abrasion are efficient in improving the bond strength of resin nanoceramic and resin-based materials [25,26]. HF etching is recommended for etchable ceramics, in order to enhance surface roughness, wettability and microretention and to reveal hydroxyl groups, which favors the chemical bonding with monomers [27].

Studies reported that the effect of HF etching on the repair bond strength for resin nanoceramics was not as high as that of sandblasting and tribochemical silica airborne particle abrasion, but more similar for hybrid ceramics [28]. Other investigations show that for the resin nanoceramics the treatment with Al_2_O_3_ was statistically higher than that with CoJet, followed by HF. For the polymer infiltrated ceramics (Vita Enamic), the airborne-particle abrasion techniques (Al_2_O_3_ or CoJet) have been reported to be ineffective and the best results were obtained with HF (9%, 60 s) followed by silane application [29,30]. Another study concluded that surface blasting followed by one layer of glaze is the best method to condition Vita Enamic [31]. Another study concluded that using 5% hydrofluoric acid for aproximatly 60 s is one of the best treatments in order to increase adhesion of the characterization layer to hybrid ceramic [32].

The investigations of resin matrix ceramic materials within this study, related to the conditioning method led to a very strong correlated conditioning behavior of resin nanoceramic materials, like Lava, Cerasmart and Shofu HC samples. For these materials the microroughness decreased thus sandblasting > tricochemical conditioning > etching, with a significant variation between sandblasting and etching. Previous materials are moderate correlated with Hyramic, another resin ceramic material, to which in addition sandblasting and tribochemical conditioning are close together and are moderate negative correlated to Enamic, a polymer infiltrated ceramic material, where etching is more efficient, resulting that all the three investigated conditioning methods lead to close microroughness values.

The topography of the surfaces was investigated by three-dimensional image generations of the surface samples, indicating visible grain boundaries after conditioning, for all materials. Nanoroughness values were strong correlated for Lava and Hyramic, with values as following: tribochemical conditioning > sandblasting > etching, but the values were not significantly different, valid for other materials as well. Significant higher values were recorded just for sandblasting, related to etching, for the nanoamplitude of heights.

In the literature the most frequent investigated CAD-CAM polymer infiltrated ceramic material was Vita Enamic (VITA Zahnfabrik), and the most frequent investigated resin nanoceramics were Lava Ultimate (3M ESPE) and Cerasmart (GC America). Among the types of surface-conditioning methods airborne-particle abrasion techniques with Al_2_O_3_ (50 µm) and CoJet 30 µm (dry and wet), grinding with a rotary instrument or, conditioning with HF (10%) followed by silane application, adhesive systems, or combinations of these techniques were used. The studies compared different treatments in the same material and found different results [33,34].

Other studies investigated the surface conditioning for adhesive repairs, in order to obtain optimal conditioning, related to surface finishing after glazing and associated with the best optical properties. Although various repair systems based on different conditioning protocols are available on the dental market, it is challenging for clinicians to choose the most appropriate system that would provide a reliable outcome [35]. Likewise, the optimal surface processing is a challenge for these newly developed materials and for which in vivo tests do not keep up.

After surface finishing, all the studied samples registered R_a_ values below 0.2 μm, generally accepted to be the cut-off value [36,37]. R_a_ and R_z_ parameters describe the surface roughness of the samples at a micro level, and S_a_ and S_y_ correspond to nanoroughness. In order to have a complex view of the samples’ behavior, micro and nanoroughness should be evaluated.

Polished, respective polished and glazed surfaces show significant lower microroughness values for Enamic and Shofu HC materials, compared to sandblasted or tribochemical conditioned and glazed ones. Finishing by polishing was proved to be a good choice for all materials taken into consideration, polishing and glazing likewise, excepting Hyramic. It could be concluded that for Enamic and Shofu HC sandblasted or tribochemical conditioned and glazing and for Hyramic polishing and glazing are not the best options, even surface roughness values are within clinically acceptable limits. Thereby the second null hypothesis “there are no differences in surface characteristics between different finishing protocols” was also rejected.

The investigations on nanolevel using AFM analyses demonstrated higher S_a_ and S_y_ values for polished surfaces for Enamic, Cerasmart and Hyramic samples, which indicates the choice of glaze, associated with the adequate conditioning method. For the other finishing methods S_a_ values are similar and between 0.52 and 3.80 nm, and for S_y_ between 6.57 and 85.22 nm.

Previously problems related to the sealants consist of low abrasion resistance, weak retention to the applied material, poor surface quality, and microcracks [38,39]. Further studies should be performed in order to investigate if surface conditioning before glazing increases the bond strength between the resin-composite CAD-CAM and the sealant, after aging procedures because resin-composite are expected to be hydrophilic [40,41,42,43,44,45,46,47]. In connection with supposed color alterations, a subsequent challenge is to evaluate the changes in optical properties of resin-composite ceramics after aging, related to surface conditioning and finishing. The study limitations were that only one type of glaze was used and that it was held in vitro and not in vivo.

## 5. Conclusions

Within the limitations of the study given the small number of developed resin-composite materials, the following conclusions can be drawn:

1. Related to the conditioning method, Lava, Cerasmart and Shofu HC samples demonstrated a very strong correlated conditioning behavior.
2. For all tested conditioning and finishing methods, resin-composite surface roughness values were within clinically acceptable limits.
3. Finishing by polishing was proved to be a good choice for all materials taken into consideration.
4. For Enamic and Shofu HC sandblasting or tribochemical conditioning and glazing and for Hyramic polishing and glazing are not the best options, related to nanoroughness values, even microroughness values are within clinically acceptable limits.

## Figures and Tables

**Table 1:** Composition and manufacturer specifications of tested materials [20,21].

Material	Type	Monomer	Filler	Manufacturer
Vita Enamic (E)	Resin composite	UDMA, TEGDMA	Feldspar ceramic enriched with aluminum oxide 86%	VITA Zahnfabrik, Bad Säckingen, Germany
Lava Ultimate (L)	CAD/CAM resin composite	Bis-GMA, UDMA, Bis-EMA, TEGDMA	SiO _2_, ZrO_2_, aggregated ZrO_2_/SiO_2_ cluster 80%	3M ESPE, Seefeld, Germany
Cerasmart (C)	CAD/CAM resin composite	UDMA, DMA	Silica, barium glass 71%	GC Corporation, Tokyo, Japan
Shofu HC (S)	CAD/CAM resin composite	UDMA, TEGDMA	Silica, silicate, zirconium silicate 61%	Shofu, Kyoto, Japan
Hyramic (H)	CAD/CAM resin composite	Resin Polymers	Inorganic Filler 55–85%	Upcera, Liaoning, China

**Table 2:** *p*-Values related to conditioning method, after conditioning.

*p*-Values	R_a_	R_z_	S_a_	S_y_
c-s	0.071	0.061	0.664	0.750
c-e	0.068	0.089	0.275	0.391
s-e	0.035	0.046	0.053	0.039

s (sandblasted), c (tribochemical silica airborne particle abrasion), e (etching).

**Table 3:** Correlation factor values related to conditioning method, after conditioning.

Spearman Correlation Factor	R_a_	R_z_	S_a_	S_y_
c-s	0.500	0.300	0.300	0.000
c-e	0.500	0.100	0.100	−0.400
s-e	0.000	0.100	0.900	0.900

s (sandblasted), c (tribochemical silica airborne particle abrasion), e (etching).

**Table 4:** *p*-Values related to material, after conditioning. E (Enamic), L (Lava Ultimate), H (Hyramic), S (Shofu), C (Cerasmart).

*p*-Values	R_a_	R_z_	S_a_	S_y_
E-L	0.984	0.623	0.287	0.362
E-C	0.731	0.627	0.532	0.351
E-S	0.746	0.962	0.079	0.054
E-H	0.365	0.371	0.403	0.932
L-C	0.818	0.641	0.055	0.662
L-S	0.240	0.169	0.129	0.044
L-H	0.292	0.365	0.970	0.742
C-S	0.375	0.719	0.063	0.041
C-H	0.289	0.196	0.471	0.812
S-H	0.087	0.156	0.282	0.438

**Table 5:** Correlation factor values related to material, after conditioning. E (Enamic), L (Lava Ultimate), H (Hyramic), S (Shofu), C (Cerasmart).

Spearman Correlation Factor	R_a_	R_z_	S_a_	S_y_
E-L	−0.500	−0.500	−0.500	−0.500
E-C	−0.500	−0.500	−1.000	1.000
E-S	−0.500	−0.500	0.500	0.500
E-H	−1.000	−1.000	−0.500	−0.500
L-C	1.000	1.000	0.500	−0.500
L-S	1.000	1.000	0.500	0.500
L-H	0.500	0.500	1.000	1.000
C-S	1.000	1.000	−0.500	0.500
C-H	0.500	0.500	0.500	−0.500
S-H	0.500	0.500	0.500	0.500

**Table 6:** *p*-Values related to conditioning method, after finishing.

*p*-Values	R_a_	R_z_	S_a_	S_y_
cg-sg	0.073	0.054	0.964	0.803
cg-eg	0.158	0.119	0.839	0.648
cg-p	0.004	0.007	0.069	0.033
cg-pg	0.188	0.123	0.588	0.764
sg-eg	0.882	0.309	0.529	0.702
sg-p	0.086	0.219	0.106	0.104
sg-pg	0.572	0.914	0.564	0.570
eg-p	0.016	0.033	0.076	0.055
eg-pg	0.339	0.177	0.113	0.325
p-pg	0.308	0.343	0.082	0.047

cg (tribochemical silica airborne particle abrasion and glazed), sg (sandblasted and glazed), pg (polished and glazed), p (polished).

**Table 7:** Correlation factor values related to conditioning method, after finishing.

Spearman Correlation Factor	R_a_	R_z_	S_a_	S_y_
cg-sg	0.771	0.029	0.200	−0.257
cg-eg	0.087	0.086	−0.700	0.429
cg-p	0.886	0.543	−0.103	0.543
cg-pg	−0.261	−0.600	0.300	0.429
sg-eg	0.551	0.771	0.300	0.200
sg-p	0.771	0.543	−0.564	−0.829
sg-pg	0.319	0.600	−0.200	−0.829
eg-p	−0.058	0.771	0.103	0.200
eg-pg	0.397	0.371	0.000	−0.257
p-pg	0.029	−0.200	0.872	0.771

cg (tribochemical silica airborne particle abrasion and glazed), sg (sandblasted and glazed), pg (polished and glazed), p (polished).

**Table 8:** *p*-Values related to material, after finishing. E (Enamic), L (Lava Ultimate), C (Cerasmart), S (Shofu), H (Hyramic).

*p*-Values	R_a_	R_z_	S_a_	S_y_
E-L	0.093	0.081	0.342	0.307
E-C	0.052	0.029	0.110	0.263
E-S	0.280	0.223	0.402	0.322
E-H	0.632	0.621	0.606	0.219
L-C	0.770	0.174	0.438	0.447
L-S	0.083	0.504	0.017	0.447
L-H	0.275	0.126	0.275	0.186
C-S	0.080	0.163	0.530	0.494
C-H	0.346	0.081	0.139	0.100
S-H	0.454	0.783	0.322	0.186

**Table 9:** Correlation factor values related to material, after finishing. E (Enamic), L (Lava Ultimate), C (Cerasmart), S (Shofu), H (Hyramic).

Spearman Correlation Factor	R_a_	R_z_	S_a_	S_y_
E-L	0.051	0.500	0.100	−0.205
E-C	0.300	0.200	1.000	0.900
E-S	0.900	0.400	0.100	−0.300
E-H	−0.700	−0.400	0.400	0.300
L-C	0.667	0.100	0.100	−0.103
L-S	0.410	0.700	1.000	0.359
L-H	0.410	0.300	−0.800	−0.872
C-S	0.400	0.400	0.100	−0.600
C-H	−0.300	−0.600	0.400	0.400
S-H	−0.400	−0.300	−0.800	−0.700

## Data Availability

The data presented in this study are available on request from the corresponding author.
